# Cardiometabolic Health: Key in Reducing Adverse COVID-19 Outcomes

**DOI:** 10.5334/gh.879

**Published:** 2020-08-19

**Authors:** Rajiv Chowdhury, Kim R. van Daalen, Oscar H. Franco

**Affiliations:** 1 Cardiovascular Epidemiology Unit, Department of Public Health and Primary Care, Strangeways Research Laboratory, Cambridge, UK; 2 University of Bern, Institute of Social and Preventive Medicine (ISPM), Bern, CH

**Keywords:** COVID-19, cardiometabolic health, public health

## Abstract

Whilst current public health measures focused on good hygiene practices and limiting person-to-person transmission contribute effectively in managing the COVID-19 pandemic, they will not prevent all individuals from becoming infected. Thus, it is of importance to explore what individuals could do to mitigate adverse outcomes. The value of beneficial health behaviours and a healthy lifestyle to improve immune functioning and lower adverse consequences of COVID-19 are increasingly being emphasized. Here we discuss seven key health behaviours and corresponding recommendations that may assist in reducing unfavourable COVID-19 outcomes.

## Healthy Lifestyle: Key in Reducing Adverse COVID-19 Outcomes

The novel coronavirus disease 2019 (COVID-19) has been declared a public health emergency of international concern with more than 17 million confirmed cases [1]. Without effective treatment or vaccines, strategies to control COVID-19 are focused on non-pharmaceutical interventions such as good hygiene practices and limiting person-to-person transmission. Whilst these measures contribute effectively in managing the pandemic, they will not prevent all individuals from becoming infected. This is particularly relevant as it is unclear for how long preventive measures can be maintained, especially in low- and middle-income countries (LMICs), and as several countries may prematurely be easing restrictions triggering secondary peaks in COVID-19 cases. Thus, it is of importance to explore what individuals could do to mitigate adverse outcomes.

**Figure 1:**
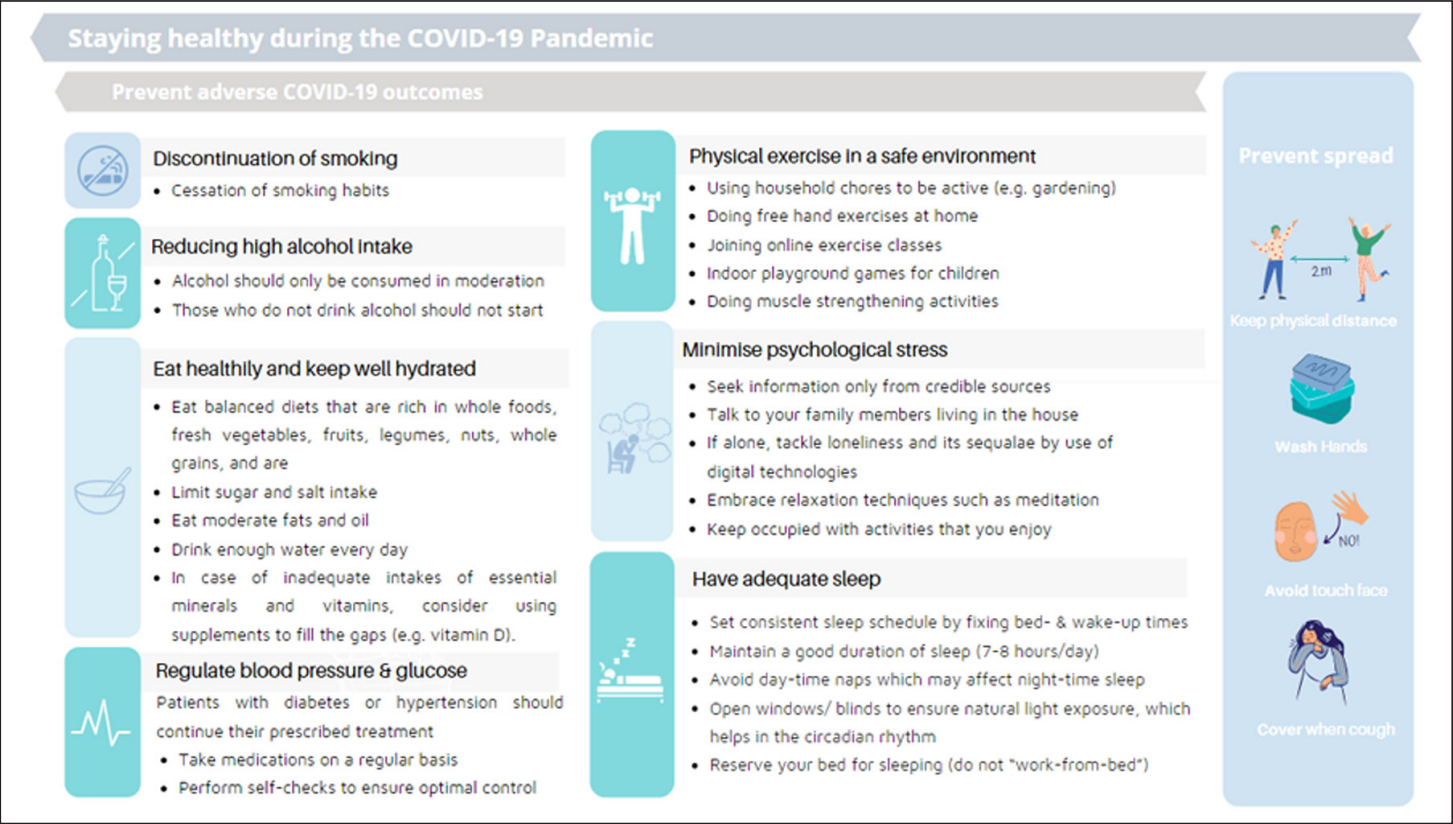
Staying healthy during the COVID-19 Pandemic.

Importantly, people with a weakened immune system or cardiorespiratory functioning are more likely to be infected with pathogens and develop complications. Accordingly, disproportionately high case-severity and case-fatality among elderly and individuals with pre-existing conditions, including cardiovascular disease, are observed among COVID-19 patients. This is also observed in relatively younger patients with co-morbid conditions or detrimental risk profiles – population characteristics common in many low-income populations [2]. Similar findings have been found during other epidemics and pandemics. For example, age and comorbidities (e.g., diabetes, CVD) were consistently found to be independent significant predictors of adverse outcomes in viruses impacting the respiratory system such as SARS [3,4,5], MERS [6], and H1N1 influenza [7]. Consequently, the value of beneficial, healthy behaviours and lifestyle to improve immune functioning and lower adverse consequences of COVID-19 are increasingly being emphasized. Here we discuss seven key health behaviours that may assist in reducing unfavourable COVID-19 outcomes whilst having important co-beneficial impacts on non-communicable disease prevention (Figure 1).

First, *regulation of cardiometabolic disease*. Cardiometabolic comorbidities are associated with adverse COVID-19 outcomes. However, many patients with hypertension or diabetes mellitus, especially in resource-poor countries, demonstrate poor adherence to essential medications [8]. Furthermore, as it was suggested that ACE inhibitors and angiotensin receptor blockers have potentially harmful effects on COVID-19 outcome, individuals have been discontinuing treatments. Yet, thus far, no studies recommend discontinuing or altering treatment. Clinical societies strongly recommend individuals with a known history of the cardiometabolic disease to regulate their blood pressure and/or glucose levels by continuing prescribed treatments including, e.g., glucose self-checks and taking medications [9].

Second, *the discontinuation of smoking*. Whilst smoking remains one of the most important preventable risk factors for premature death, recent evidence suggests a lack of association between smoking and adverse COVID-19 outcome [10]. This could partly be explained by high case-fatality among elderly patients with multiple comorbidities tending to be non- or former smokers due health reasons. Nonetheless, smoking results in comorbidities that have been associated with poor COVID-19 outcomes, including cardiac events. Furthermore, smoking is known to be associated with viral infections and severity (e.g., influenza) as smokers are more likely to have poorer lung function/capacity [11], contract microbial diseases (through structural changes in the respiratory tract, decreased immunity) [12], and perform repetitive hand-to-mouth movements. Considering this, recommendations advocate for an immediate cessation of smoking habits to prevent detrimental COVID-19 consequences.

Third, a *healthy, balanced diet*. Nutrition and hydration remain cornerstones for optimal health. Various micro- and macro-nutrients play potentially crucial roles in immunity [13] (e.g., zinc regulates cell division [14], vitamin D is needed for natural killer cell function [15], and arginine is essential for nitric oxide generation by macrophages [16]), whilst deficiencies and prolonged undernutrition impair immune function [13]. Hence balanced rich in whole foods, fresh vegetables, fruits, legumes, nuts, whole grains, adequate hydration, and are low in sugar and salt are recommended [17]. Additionally, some evidence suggests that nutritional supplements might be beneficial in preventing acute respiratory tract infections and adverse outcomes, including COVID-19. For example, it has been recommended for people at risk of influenza and/or COVID-19 to consider taking 10,000 IU/d of vitamin D_3_ for a few weeks to raise 25(OH)D concentrations, followed by 5000 IU/d [18]. Furthermore, supplementation of vitamin A, D, Zinc, Selenium, as well as the use of several nutraceuticals and probiotics may be able to enhance the prevention and treatment of COVID19 infections [19]. However, appropriate caution should be taken to avoid the potential risk of overdose, and natural food sources are preferred. Those at risk of malnutrition or those malnourished should take extra precautionary care, ideally assisted by a trained dietician [19].

Fourth, *safe physical exercise*. Regular physical activity or exercise improves immune regulation whilst delaying and decreasing the incidence of infections and non-communicable diseases (e.g., cancer, CVD, chronic inflammatory disorders) [20]. Furthermore, it stimulates healthy aging by reducing risk on elements of frailty (e.g., impaired mobility), sarcopenia, and dementia [21]. Acute exercise (moderate-to-vigorous, up to 60 min/day) is considered as important immunoregulator, supporting optimal exchanges of distinct, highly active immune cell subtypes between tissues and circulation [22]. Nevertheless, prolonged home-stays result in increased sedentary behaviour and lack of physical activity, leading to chronic health conditions, anxiety, and lower immunity. It is therefore advised to keep physically active, whilst following safety precautions. There is a plethora of ways to keep physically active. Examples recommended, but are not limited to, include: 1) using household chores to be active (e.g., cleaning tasks), 2) freehand exercises at home, 3) joining online exercise classes, 4) muscle-strengthening activities if feasible, and 5) indoor playground games for children [23]. A multicomponent exercise program (aerobic resistance, balance, coordination, and mobility training) is considered most adequate for older people [21]. It is imperative that all safety precautions and infection control measures should be followed when undertaking any form of physical exercise at or around the home [24]. For instance, the right activity should be chosen to maintain physical distancing, reduce the risk of injury, and exercise should be avoided if there is a symptom (e.g., fever, cough or breathing difficulty). Furthermore, public gyms or pools are best avoided.

Fifth, *minimize stress*. Physiological stress may increase susceptibility to communicable diseases (including respiratory infections) due to compromised immunity and increased inflammatory reactivity resulting from, e.g., modulation of the hypothalamic-pituitary-adrenal axis in response to stress [25]. Such negative impacts of stress [26,27] on infection and immunity are very pertinent to the COVID-19 pandemic, as entire communities are now in lockdown, the economic future is uncertain, and people are deeply concerned about the health and wellbeing of themselves and their loved ones. Furthermore, large scale disasters are almost always accompanied by increases in depression, posttraumatic stress disorder, substance use disorders, domestic violence, and a range of other mental health impacts [28]. Worryingly, a significant negative association appears to exist in people with psychological stress and their antibody responses to influenza vaccination [29]. Reducing, psychosocial stress might be beneficial to successfully fight off COVID-19. Several proactive measures to minimize the effects of stress are recommended: 1) develop and implement a routine to ensure continuity and structure, 2) take breaks from reading or listening to distressing news (or misinformation) that are circulating through social or other media, 3) seek information only from credible sources; 4) talk to your family members living in the house; 5) if you live alone, tackle loneliness and its sequalae by e.g. the use of digital technologies, 6) embrace relaxation techniques such as meditation or prayers; 7) keep yourself occupied with activities that you enjoy [30,31].

Sixth, *maintaining adequate sleep*. Sleep is essential to health and effective immune functioning (e.g., modulation cytokines levels, cell subpopulations) [32,33]. Conversely, sleep deprivation, and rapid eye movement sleep deprivation can weaken immunity and increase susceptibility to viral infections [33]. This has previously been shown for e.g., influenza virus [34]. Furthermore, sleep heightens mood and optimizes energy levels [35], and sleep improves overall brain function and mental health (e.g., reducing stress and anxiety) [35]. Therefore, while no direct evidence on sleep deprivation and the risk of adverse COVID-19 outcomes currently exist, it seems reasonable that consistent, high-quality sleep may be immune-supportive. Recommendations include: 1) set a consistent sleep schedule by fixing the bedtime and wake-up times, 2) maintain good duration of sleep (7–8 hours/day), 3) avoid day-time naps which may affect night-time sleep, 4) open windows/blinds to ensure natural light exposure, which supports circadian rhythm, 5) reserve your bed for sleeping (do not ‘work-from-bed’), 6) limit the exposure to digital screens specially before sleep [36].

Seventh, *reducing high alcohol intake*. This March, various people died after drinking industrial-strength alcohol, based on a false belief that it would protect them from COVID-19. Inversely, associations with heavy alcohol drinking and adverse infection outcome have been reported throughout history. Epidemiological studies found associations between alcohol abuse and viral infections, including HIV-AIDS [37], hepatitis C [38], and community-acquired pneumonia [39]. Excess alcohol disrupts adaptive immunity by affecting T lymphocytes balance, T-cell functioning, and peripheral B cells [40]. Reducing high alcohol intake may be favourable for COVID-19 susceptibility and outcomes. In line with the World Health Organization guidelines, it is recommended that alcohol intake should be eliminated or only be consumed in moderation. Also, those who do not drink alcohol should not start drinking [41].

Although these recommendations could improve immune function and overall health, implementation faces challenges. First, behaviour has proven difficult to change, even after facing detrimental disease, and it is unclear whether COVID-19 concerns would suffice to motivate individuals to change behaviour [42]. Second, built and physical environment can limit health behaviour implementation. To illustrate, people living in food deserts, with limited access to healthy foods, often have high caloric diets low in nutritional value. Poor urban planning (e.g., limited infrastructure compactness) and overcrowding can lead to lower means to do physical activity [43]. Third, food availability and access to food (especially fresh foods) may be limited. Fourth, prevention and treatment services have been severely disrupted. Hence, medication availability might be affected by lockdown measures and the concentration of healthcare systems on pandemic management, limiting medication adherence. Furthermore, many populations already have poor access to effective, equitable healthcare, including cardiometabolic medications. Finally, inequalities in economic, cultural, and social resources my shape inequalities in abilities to adapt health behaviours, having potential to exacerbate health inequalities [44]. This is particularly problematic as pandemics are most destructive in vulnerable populations.

While being conscious of implementation challenges, proposed actions provide guidance on health behaviours improving immune and cardiorespiratory function that may reduce adverse COVID-19 outcomes. However, it should be noted that concerted efforts through multilateral cooperation and integrative efforts, including consideration of the social and environmental factors of inequalities and societal solidarity are essential to facilitate their effective implementation. This includes, for example, ensuring mechanisms in place for reporting and intervention of domestic violence and mental health system preparation for the inevitable consequences precipitated by the pandemic [30]. Short-term and long-term benefits of a healthy lifestyle may be enormous and expand beyond the current pandemic, benefiting individual, societal and planetary health.

## Author Information

Oscar Franco, MD MSc. DSc. PhD., is a professor of Epidemiology and Public Health and director of the Institute of Social and Preventive Medicine at the University of Bern. Rajiv Chowdhury, MD MPhil PhD., is a reader in Global Health at the University of Cambridge. Kim van Daalen, BSc. MPhil., is a PhD student at the University of Cambridge.
